# A Shared Population of Epidemic Methicillin-Resistant *Staphylococcus aureus* 15 Circulates in Humans and Companion Animals

**DOI:** 10.1128/mBio.00985-13

**Published:** 2014-05-13

**Authors:** Ewan M. Harrison, Lucy A. Weinert, Matthew T. G. Holden, John J. Welch, Katherine Wilson, Fiona J. E. Morgan, Simon R. Harris, Anette Loeffler, Amanda K. Boag, Sharon J. Peacock, Gavin K. Paterson, Andrew S. Waller, Julian Parkhill, Mark A. Holmes

**Affiliations:** ^a^ Department of Veterinary Medicine, University of Cambridge, Cambridge, United Kingdom; ^b^ Wellcome Trust Sanger Institute, Hinxton, United Kingdom; ^c^ Department of Genetics, University of Cambridge, Cambridge, United Kingdom; ^d^ Royal Veterinary College, University of London, North Mymms, Hertfordshire, England; ^e^ Department of Clinical Medicine, University of Cambridge, Cambridge, United Kingdom; ^f^ School of Biological, Biomedical and Environmental Sciences, University of Hull, Hull, United Kingdom; ^g^ Centre for Preventive Medicine, Animal Health Trust, Kentford, Newmarket, Suffolk, United Kingdom

## Abstract

Methicillin-resistant *Staphylococcus aureus* (MRSA) is a global human health problem causing infections in both hospitals and the community. Companion animals, such as cats, dogs, and horses, are also frequently colonized by MRSA and can become infected. We sequenced the genomes of 46 multilocus sequence type (ST) 22 MRSA isolates from cats and dogs in the United Kingdom and compared these to an extensive population framework of human isolates from the same lineage. Phylogenomic analyses showed that all companion animal isolates were interspersed throughout the epidemic MRSA-15 (EMRSA-15) pandemic clade and clustered with human isolates from the United Kingdom, with human isolates basal to those from companion animals, suggesting a human source for isolates infecting companion animals. A number of isolates from the same veterinary hospital clustered together, suggesting that as in human hospitals, EMRSA-15 isolates are readily transmitted in the veterinary hospital setting. Genome-wide association analysis did not identify any host-specific single nucleotide polymorphisms (SNPs) or virulence factors. However, isolates from companion animals were significantly less likely to harbor a plasmid encoding erythromycin resistance. When this plasmid was present in animal-associated isolates, it was more likely to contain mutations mediating resistance to clindamycin. This finding is consistent with the low levels of erythromycin and high levels of clindamycin used in veterinary medicine in the United Kingdom. This study furthers the “one health” view of infectious diseases that the pathogen pool of human and animal populations are intrinsically linked and provides evidence that antibiotic usage in animal medicine is shaping the population of a major human pathogen.

## IMPORTANCE

Methicillin-resistant *Staphylococcus aureus* (MRSA) is major problem in human medicine. Companion animals, such as cats, dogs, and horses, can also become colonized and infected by MRSA. Here, we demonstrate that a shared population of an important and globally disseminated lineage of MRSA can infect both humans and companion animals without undergoing host adaptation. This suggests that companion animals might act as a reservoir for human infections. We also show that the isolates from companion animals have differences in the presence of certain antibiotic resistance genes. This study furthers the “one health” view of infectious diseases by demonstrating that the pool of MRSA isolates in the human and animal populations are shared and highlights how different antibiotic usage patterns between human and veterinary medicine can shape the population of bacterial pathogens.

## INTRODUCTION

*Staphylococcus aureus* is part of the natural microbiota of humans and animals but has the potential to cause a broad spectrum of infections. The emergence and spread of methicillin-resistant *S. aureus* (MRSA) in both hospital and community settings pose a major threat to global health. Since the initial description in 1961, MRSA has spread globally with a small number of specific clones, including multilocus sequence types 239 (ST239), ST22, and ST8, causing the majority of the burden of disease (1, 2). Since the late 1990s, the role that both livestock and companion animals play as reservoirs and vectors for transmission of MRSA has become clearer (3–5). This relationship seems to be bidirectional, with human origins for the ST5 poultry lineage (6) and clonal complex 398 (CC398) originally infecting humans, jumping to livestock, and then back to humans (7, 8). More recently a number of “multihost” MRSA lineages with a novel *mecA* gene homologue, named *mecC*, were identified, which are capable of colonizing and infecting a broad range of mammalian and avian species (9–11). Companion animals, such as cats, dogs, and horses, are also frequently colonized by MRSA and can become infected (11–13). In dogs, reported MRSA carriage rates range from 0.7% in Portugal (14), to 2.3 to 9% in the United Kingdom (15, 16), and up to 20% in an outbreak in Canada (17). The prevalence of MRSA colonization of cats appears to be lower than that for dogs, with reported carriage rates of 0 to 4% (18), including 1.4% in Portugal (14) and 1.48% in the United Kingdom (13). Risk factors for MRSA infection in companion animals include contact with human MRSA carriers, the number of courses of antimicrobials received, length of time spent in veterinary clinics, and use of surgical implants (12, 15). MRSA lineages isolated from companion animals generally match the dominant lineages found in the human populations in the same geographical area: ST22 (epidemic MRSA 15 [EMRSA-15]) in the United Kingdom (19), Germany (20, 21), Portugal (22), and ST59/ST239 in China (23, 24). Molecular epidemiology using pulsed-field gel electrophoresis (PFGE) and *spa* typing has found that human and companion isolates are indistinguishable, suggesting transmission between humans and companion animals (25–29). Furthermore, a small number of studies have identified companion animals as the likely source for human MRSA infections (3, 28, 30). In the United Kingdom, ST22 (EMRSA-15) makes up the bulk of hospital-acquired cases of MRSA (31). A recent detailed phylogenomic study of the ST22 lineage demonstrated that it is likely to have emerged in the United Kingdom in the 1980s and then spread globally, with its initial success probably driven by the acquisition of fluoroquinolone resistance at a time of increasing fluoroquinolone usage (32). A single-locus variant of ST22 (ST2371) with Pantón-Valentine leukocidin (PVL) was recently tracked using whole-genome sequencing as spreading from a hospital outbreak into the community, demonstrating the highly transmissible nature of this clone (33). In this study, we sequenced the genomes of 46 isolates from companion animals (4 feline and 42 canine) from a collection of ST22 MRSA isolates from the United Kingdom isolated between 2003 and 2007. We compared these isolates to the recently published ST22 phylogeny (32) in order to understand the phylogenetic relationship between isolates infecting humans and companion animals. This showed that a shared population of ST22 isolates infects humans and companion animals. This study confirms the extended host spectrum of ST22 isolates, which is potentially a key factor contributing to the success of this lineage.

## RESULTS

### Phylogenetics of companion animal isolates in comparison to human isolates.

**FIG 1:**
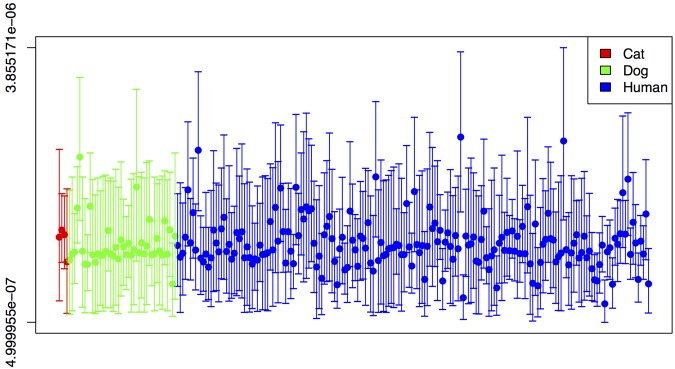
Rates of evolution on the terminal branches of the dated phylogeny. Points represent median estimates, and lines represent 95% highest posterior density (HPD) estimates of substitution rate (substitutions per site per year) for each terminal branch in a maximum clade consensus tree. Branches are colored by the host state associated with that branch: red for cats, green for dogs, and cyan for humans.

**FIG 2:**
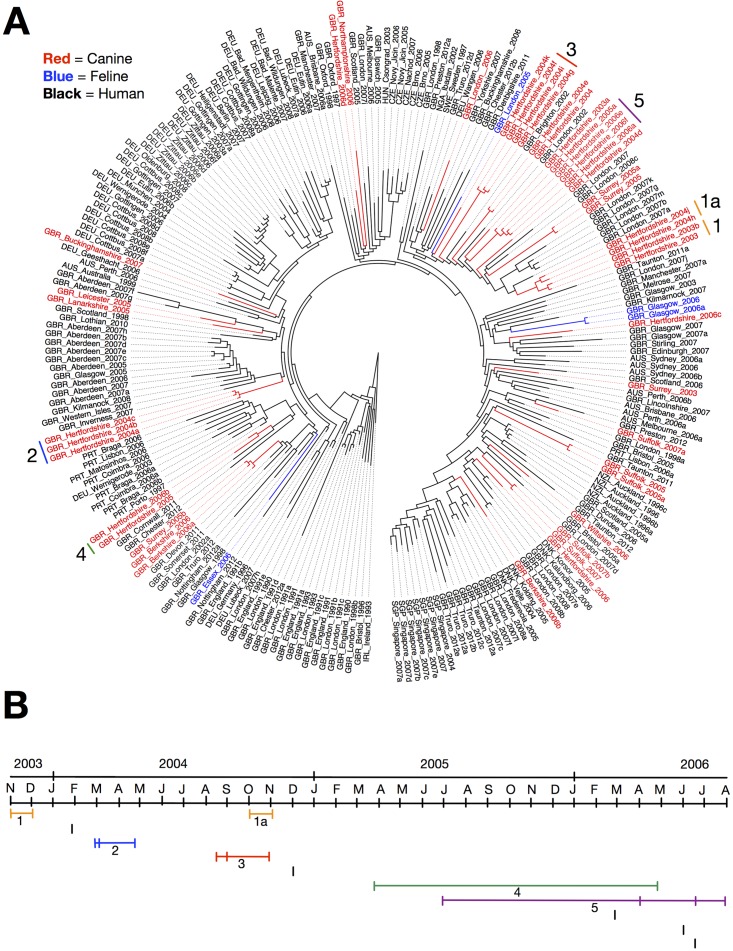
Phylogenetic relationships between human and companion animal isolates. (A) A maximum likelihood tree generated from SNPs in the core genome is shown. The tree is rooted in isolate IRL_Ireland_1993 as an outgroup (previously identified as basal to the EMRSA-15 clade by Holden et al. [32]). Isolates and branches marked in red, blue, and black are from dogs, cats, and humans, respectively. Closely related isolates from the Royal Veterinary Hospital, Hertfordshire, United Kingdom, with available temporal information are highlighted and numbered based on date of isolation. (B) Time line of isolates in the Royal Veterinary Hospital, Hertfordshire, United Kingdom. Individual marks denote an isolate. Numbering denotes clades highlighted in the phylogeny.

**FIG 3:**
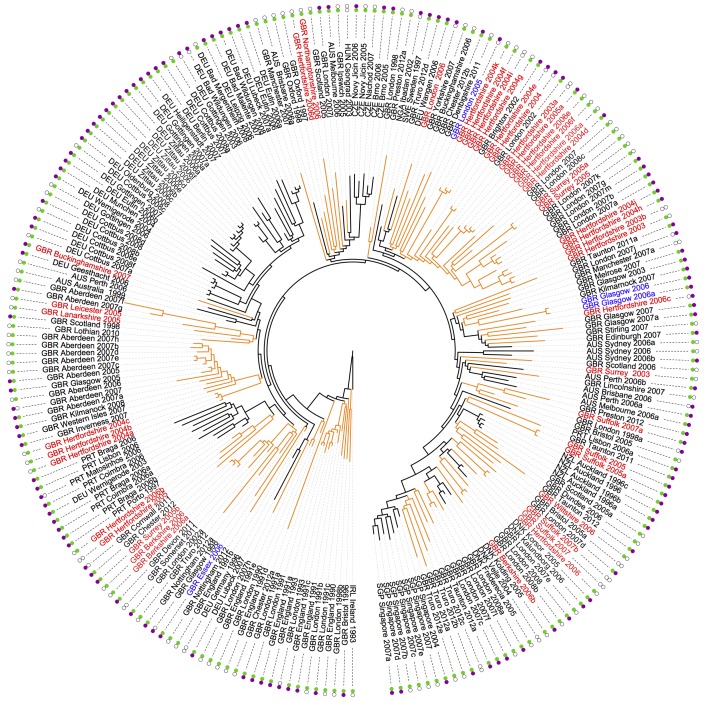
Presence and absence of ϕSa3 phage (*sak*, *chips*, and *scn*) and *erm*(*C*) in isolates in the ST22 phylogeny. Figure shows a maximum likelihood tree generated from SNPs in the core genome, the tree is rooted in isolate IRL_Ireland_1993 as an outgroup. Isolates marked in red, blue, and black are from dogs, cats, and humans, respectively. Branches and clades of isolates from the United Kingdom are marked in orange. The presence and absence of ϕSa3 phage and *erm*(*C*) are indicated by the green and purple dots, respectively.

To investigate the relationship between ST22 isolates from companion animals and humans, we sequenced the genomes of 46 ST22 isolates from companion animals (42 canine and 4 feline) isolated between August 2003 and August 2007 (see Table S1 in the supplemental material). The isolates were from two large veterinary hospitals (The Royal Veterinary College, Herefordshire, United Kingdom [24 isolates] and The Animal Health Trust, Suffolk, United Kingdom [5 isolates]), and a number of smaller veterinary practices throughout the United Kingdom (17 isolates). The companion animal isolates were from infections similar to those associated with ST22 in humans, with the majority of isolates coming from wound infections (including surgical site infections) (21) or skin and soft tissue infection (SSTI) (7). The collection also included isolates from urine (4), cerebrospinal fluid (CSF) (2), and nasal wash or discharge (2), and one isolate each from a bloodstream, heart valve, and joint infection. Only one isolate was from nasal carriage, and for six isolates, the clinical source was unknown. An additional 22 human CC22 isolates sequenced as part of other studies were also included in the analysis (34). The sequences were mapped against the ST22 reference genome HO 5096 0412 and then combined with the ST22 isolates previously reported by Holden et al. (32). Single nucleotide polymorphisms (SNPs) in the core genome were then used to reconstruct the phylogenetic relationships between the isolates. The substitution rate across the core genome under a constant population size model was estimated as 1.47 × 10^−6^ per nucleotide site per year (95% Bayesian credible interval, 1.34 × 10^−6^ to 1.60 × 10^−6^), and for an exponentially growing population, it was estimated as 1.48 × 10^−6^ (95% Bayesian credible interval, 1.36 × 10^−6^ to 1.60 × 10^−6^), similar to that previously reported for ST22 (32). No statistically significant difference was seen between the substitution rates for companion animal and human isolates (Fig. 1). Analysis of the phylogeny revealed that all of the companion animal isolates belonged to the previously described epidemic MRSA 15 (EMRSA-15) pandemic clade (ST22-A2 in reference 32) (Fig. 2A). The companion animal isolates were broadly distributed throughout the EMRSA-15 pandemic clade and, with one or two exceptions, clustered together with human isolates in United Kingdom-specific clades (Fig. 2A and 3). In all cases, human isolates were basal to those from companion animals, indicating that the evolutionary origin of the companion animal isolates was likely to be human. One particularly large clade of isolates contained human, cat, and dog isolates from three different veterinary practices in London and the southeast of England (see isolates in and around clades 1, 3, and 5 in Fig. 2A and 3).

### Isolates from large veterinary hospitals cluster together.

In general, the isolates from the larger veterinary hospitals clustered together on the phylogenetic tree, while those from individual veterinary practices were not clustered with other companion animal isolates (see isolates from the Royal Veterinary College [RVC]) (isolates labeled “Hertfordshire” in Fig. 2A) and from the Animal Health Trust (AHT) (isolates labeled “Suffolk” in Fig. 2A). This was confirmed by randomization tests for significant clustering on the tree (isolates from large hospitals, *P* = 0.011; isolates from individual veterinary practices, *P* = 0.929). The RVC is situated in the southeast of England just outside London, and the majority of the isolates from the RVC (16 of 24 isolates) were also part of the large clade (described above) containing both human and companion animal isolates from London and the southeast (see isolates in and around clades 1, 3, and 5 in Fig. 2A). The phylogeny suggests that the RVC isolates were drawn from a population of EMRSA-15 isolates that was circulating widely throughout the human population of London and the southeast. Further analysis, focusing on isolates from the RVC, showed that multiple distinct clades with two or more closely related isolates separated by a small number of SNPs were circulating in the RVC between November 2003 and August 2006 (clades 1 to 5 in Fig. 2A). In two cases, in clades 2 and 3, three closely related isolates from different animals were isolated within ~2 months of each other (Fig. 2A and B), while isolates from clades 1, 4, and 5 were isolated from animals over a longer time periods (~6 to 12 months) (Fig. 2B).

Within each clade, isolates from a range of infections were present, demonstrating the ability of highly related isolates to cause a broad spectrum of disease in companion animals, as is the case in humans. In addition to the clusters of related isolates, a number of individual phylogenetically distinct isolates were also associated with infections in the RVC throughout the same period (Fig. 2A and B). A similar picture was seen in isolates from the other veterinary hospital in the study, the AHT, where two clades of closely related isolates were identified (isolates GBR_Suffolk_2005/a and GBR_Sufflok_2007/b in Fig. 2A). Phylogenetic analysis also identified a case of chronic infection or reinfection. Two wound isolates from the same cat taken ~20 days apart (GBR_Glasgow_2006 and GBR_Glasgow_2006a in Fig. 2), differed by only 3 SNPs, indicating that they shared a recent common ancestor. The level of diversity in these isolates is well within the observed variation of isolates in known cases of EMRSA-15 transmission (35), suggesting either a chronic wound infection or reinfection from the same source (Fig. 2A). Unfortunately, there was no further clinical information available to investigate the epidemiology.

### Comparison of virulence factors and antibiotic resistance genes between human and animal isolates.

In order to investigate if there was a genetic basis for the ability to colonize and cause disease in different hosts, we performed comparative genomic analysis on the ST22 isolates from the companion animals and human ST22 isolates. The entire collection of companion animal isolates was genotypically MRSA and harbored the SCC*mec* type IVh element as seen in the human ST22 isolates (32). All of the companion animal isolates had both of the fluoroquinolone resistance mutations in Ser80Phe in GrlA and Ser84Leu in GyrA previously described in human ST22 isolates (32). We assessed if the animal and human isolates shared the same virulence factors and antibiotic resistance genes previously identified in the human isolates (see Table S1 in the supplemental material) (32). We identified that the companion animal isolates were statistically more likely to have lost both the ϕSa3 phage (containing the human-specific immune evasion genes *sak*, *chips*, and *scin*), with 76.1% ϕSa3 positive, cf. 90.2% (Fisher’s exact test, *P* = 0.022), and the plasmid borne *erm*(C) erythromycin resistance gene, with 37% *erm*(C) positive, cf. 62% (Fisher’s exact test, *P* = 0.002) (Fig. 3). These analyses treat each isolate as an independent observation and so neglect the evolutionary history of the isolates as revealed by phylogenetic analysis. To control for phylogenetic nonindependence, we treated the host state (human versus companion animal) and the presence of the ϕSa3 phage (present versus absent) as binary traits and tested whether these traits evolved across the phylogeny in a correlated fashion. The results showed that a model with correlated evolution was not preferred to a model in which the traits evolved independently (Bayes factor 1, BayesTraits [73]), suggesting that companion animal isolates were no less likely to lose the ϕSa3 phage than would be expected by chance. However, when we applied the same test to *erm*(C), correlated evolution between host state and loss/gain of *erm*(C) had substantial support (Bayes factor 9), providing further evidence that loss of *erm*(C) was associated with isolates coming from companion animals rather than humans. This host-species-associated loss is clearly visible on the phylogenetic tree, with the clades of companion animal isolates having lost the ErmC-encoding plasmid, while closely related and basal human isolates retained this plasmid (Fig. 3). We also identified three feline isolates (GBR_Glasgow_2006a, GBR_Glasgow_2006b, and GBR_Essex_2006) with genetic rearrangements disrupting the regulatory region upstream of *erm*(C), rendering its expression constitutive and thereby conferring resistance to clindamycin (36). Resistance to clindamycin in all three isolates was confirmed by phenotypic testing. Interestingly, the clindamycin-resistant isolates included the two isolates from the same cat described previously (GBR_Glasgow_2006a and GBR_Glasgow_2006b) that only differ by 3 SNPs. Furthermore, these two isolates each had different genetic rearrangements in the leader peptide, suggesting that two separate events led to resistance to clindamycin in these closely related isolates. The chronologically first isolate from the cat (GBR_Glasgow_2006) had an insertion (IS) element inserted in the leader peptide, 66 bp upstream of the *erm*(C) start codon, while the second isolate, isolated ~20 days later, had a 58-bp deletion of the leader peptide, 69 bp upstream of the *erm*(C) start codon, (GBR_ Glasgow_2006a). The third isolate, also from a cat (GBR_Essex_2006), contained an insertion of an IS element in the leader peptide region, 67 bp upstream of the start codon of *erm*(C). In contrast, none of 22 human isolates from the United Kingdom included in this study had the same or functionally equivalent rearrangements, as had been reported previously by Holden et al. (32) for the rest of United Kingdom human ST22 isolates and as found also in a wider study of the United Kingdom population of ST22 isolates (S. Peacock, personal communication).

### No evidence of adaption to companion animals.

To further identify if the isolates infecting the companion animals had mutations that might be associated with host adaptation, we used a genome-wide association (GWAS) approach. This approach uses multivariate regression analysis to identify SNPs in the core genome that are significantly associated with host state (e.g., SNPs present or absent in companion animal versus human isolates). The analysis showed very little genetic discrimination between isolates from different hosts (see Fig. S2 in the supplemental material), suggesting that the ST22 isolates had not undergone extensive adaptation to companion animals. Five SNPs (three nonsynonymous, two synonymous) showed a more marked correlation with host state (companion animals) (see Fig. S3 and Table S3 in the supplemental material). However, ancestral state reconstruction of these SNPs across the phylogeny identified that they had each arisen only once in a single clade with both companion animal isolates and human isolates (Fig. 1 and 3, isolates around clades 1, 3 and 4). In addition, they had not been lost in human isolates from this clade (Fig. 2A). As such, it is most parsimonious to assume that the population structure alone was responsible for this correlation. Therefore, using this approach we were unable to identify any homoplasic SNPs potentially involved in host adaption to the companion animals. We also investigated pseudogenes present in the ST22 isolates in an attempt to identify any particular gene inactivations that might be associated with adaption to companion animals. However, we were unable to find any pseudogenes that had arisen more than once in companion animal isolates when taking into account the population structure (data not shown).

## DISCUSSION

This is the first high-resolution genomic study of companion animal MRSA isolates, confirming what has been previously inferred from epidemiological data and lower-resolution molecular techniques (multilocus sequence typing [MLST], *spa*, and PFGE typing) that humans and companion animals readily exchange and share the MRSA isolates from the same population (16, 21, 25). Beyond this, we identified geographical structuring of human and companion animal isolates from London and southeast England, suggesting that the same lineage was being exchanged between humans and companion animals in the local population. The widespread distribution of companion animal isolates throughout the EMRSA-15 phylogeny demonstrates that in all likelihood most EMRSA-15 isolates are capable of infecting both humans and companion animals. This ability to infect different hosts has been suggested to be an “extended-spectrum genotype” (20). A similar ability to cause infections in multiple species with limited genomic variation between strains has also been reported for ST130 (37) and CC398 (7). Speculatively, this ability to colonize and infect multiple species might be a beneficial adaption that provides EMRSA-15 isolates with an alternative pathway for transmission between human populations and that has contributed to the success of EMRSA-15, which is now the dominant lineage in much of Europe (38). Additionally, ST8 (USA300), the dominant lineage in North America, has been identified in cattle in Switzerland (39), cats and dogs in France (40), and cats, dogs, and pigs in the United States (41) and has been shown to be able to bind porcine corneocytes and persist in the nasal cavity of pigs (42). This suggests that a broad host range might be a common feature of successful *S. aureus* lineages and be characteristic of their long-term evolutionary history. Alternatively this might simply be due to a founder effect and simply reflect the predominant clones circulating in a particular geographical area.

We identified that, in a number of cases, isolates from the same veterinary hospitals clustered together, and highly related isolates were present in the same veterinary hospital over extended periods of time (~6 to 12 months). This suggests that EMRSA-15 isolates were persisting and being readily transmitted within the veterinary hospitals, as has been observed in human hospitals, suggesting that the pandemic potential of this clone is greater than generally acknowledged (33, 43). This finding is also consistent with the fact that all of the companion animal isolates in this study were from the predominately hospital-acquired EMRSA-15 (ST22-A) sublineage identified by Holden et al. rather than the predominately community-acquired broader ST22 group (32). The transmissibility of a hospital-acquired MRSA clone in a veterinary hospital setting demonstrates that MRSA prevention practices used in human medicine, such as search and destroy or blanket decolonization, may also be appropriate in veterinary practice (44). Further studies using whole-genome sequencing to investigate temporally paired human and companion isolates from veterinary hospitals and the human community are warranted to further understand the exact transmission dynamics between humans and companion animals.

A number of studies have identified genetic changes associated with adaptation of *S. aureus* to new host species (6, 7), including the most recent study of CC97 isolates jumping from cattle into humans (45). A common theme among these studies is the acquisition or loss of the β-toxin-converting phage (ϕSa3), which encodes the modulators of the human innate immune response; staphylokinase (SAK), staphylococcal complement inhibitor (SCIN), and chemotaxis inhibitory protein of *S. aureus* (CHIPS). We found no significant difference in the presence or absence of the ϕSa3 phage between human and companion animal isolates when correcting for shared evolutionary history. This lack of difference in the presence of ϕSa3 might be expected. While both CHIPS and SCIN are human specific and have significantly reduced activity against canine serum and neutrophils, respectively, SAK has been demonstrated to enhance the activation of canine plasminogen activation *in vitro*, suggesting that it might play a role in canine infections (46–48). No further loss or gain of virulence factors was associated with the isolates infecting companion animals, nor were any particular mutations (SNPs) detected in the core genome by a GWAS approach. This leads us to the conclusion that the core EMRSA-15 genome, present in isolates throughout the EMRSA-15 phylogeny, is sufficient to confer isolates with an extended host spectrum. EMRSA-15 has also been isolated from horses in Germany, United Kingdom, and Ireland (49, 50), goats in Spain (51), and a broad range of animal species, including a rabbit, parrot, turtle, and bat in Germany (52). Further studies including these isolates are needed to investigate if the same is true for isolates from diverse animal species.

One particularly interesting finding was that the companion animal isolates were significantly less likely to have maintained the plasmid-carried *erm*(C) gene, suggesting that the selective pressures for maintenance of the *erm*(C) plasmid are less in companion animals or that *erm*(C) is selected against. Previously, Harris et al. (33) reported the loss of the *erm*(C) plasmid in the CC22 isolates from two patients and in 18 of 20 colonies sequenced from a single colonized individual, suggesting this might be relatively common occurrence in the absence of selection (33, 53), as has been reported for other plasmids (54). Antibiotic resistance itself can come with significant fitness costs that can exact selective pressure elsewhere in the genome (55–57). In the United Kingdom, the use of erythromycin is rare in small animal veterinary medicine. During the period when the isolates in this study were collected (2005 to 2007), no erythromycin authorized for use in companion animals was sold in the United Kingdom (58–60). Another study by Mateus et al. in 2007 of 11 United Kingdom veterinary practices found that erythromycin usage was very rare and made up only 0.16% and 0.08% of prescriptions in dogs and cats, respectively (61). The picture in human medicine is dramatically different: 6.04 million prescriptions for erythromycin were made by general practitioners (GPs) between April 2005 and March 2007 in England alone (62). Thus, it is likely that the lack of usage of erythromycin in veterinary medicine in the United Kingdom meant there was a lack of selective pressure for the maintenance of *erm*(C) in companion animal isolates.

We also identified that three of the feline isolates had separate genetic rearrangements in the *erm*(*C*) leader peptide that are associated with the generation of clindamycin resistance. Previously, Holden et al. (32) suggested that the lack of clindamycin resistance in United Kingdom isolates from humans in comparison to isolates from other countries, including Germany and Sweden, was due to much higher clindamycin usage in these countries than in the United Kingdom. While there is very little use of lincosamides (of which clindamycin is a member) in human medicine in the United Kingdom (63), clindamycin is used widely in veterinary practice in the United Kingdom (61). A total of 2,533 kg of clindamycin authorized for use in companion animals was sold between 2005 and 2007 (58–60). Although clindamycin resistance was only present in three companion animal isolates, the fact that we identified two highly related isolates with different kinds of genetic rearrangements (IS insertion and deletion) combined with the significantly higher usage of clindamycin in veterinary practice does suggest that there might be a greater selective pressure for clindamycin resistance in veterinary settings as opposed to human health care settings.

We conclude that the intermingling of human and companion animals isolates within the EMRSA-15 pandemic clade suggests that isolates were capable of moving between the two populations, providing further support for the notion that companion animals may act as a reservoir for human MRSA infection and vice versa (64). This, combined with the findings related to difference in antibiotic resistance between human and companion animal isolates, highlights the importance of a “one health” view of infectious diseases—that the health of both human and animal populations are intrinsically linked.

## MATERIALS AND METHODS

### Isolates.

Isolates were clinical cases submitted to diagnostic laboratories at the Animal Health Trust and Royal Veterinary Hospital (some isolates referred via Compton Paddock Laboratories) between August 2003 and August 2007. The isolates were subjected to in-house multilocus sequence typing, and all ST22 isolates were selected to be included for sequencing.

### Whole-genome sequencing.

Overnight cultures were grown in tryptic soy broth (TSB) at 37°C with 200-rpm shaking. Genomic DNA was extracted from cultures by using the MasterPure Gram-positive DNA purification kit (Cambio, United Kingdom). Illumina library preparation was carried out as described by Quail et al. (65) Hi-seq sequencing was carried out following the manufacturer’s standard protocols (Illumina, Inc., United States). Nucleotide sequences of the isolates have been deposited in the Sequence Read Archive database in the European Nucleotide Archive (see Table S1 in the supplemental material).

### Antibiotic resistance testing.

Antimicrobial susceptibility testing was performed using disk susceptibility testing according to BSAC criteria (*BSAC Methods for Antimicrobial Susceptibility Testing*, version 11.1, May 2012; British Society for Antimicrobial Chemotherapy, Birmingham, United Kingdom). NCTC12493 and NCTC6571 were used, respectively, as control resistant and susceptible isolates for clindamycin.

### Phylogenetics and comparative genomics.

Fastq files for the isolates were mapped against the ST22 MRSA reference genome HO 5096 0412 (EMBL accession no. HE681097) using SMALT (http://www.sanger.ac.uk/resources/software/smalt/) in order to identify single nucleotide polymorphisms (SNPs), as previously described (32, 35). SNPs located in mobile genetic elements (MGEs) were identified and removed from the alignment to generate a core genome (regions of the chromosome not excluded when MGEs were removed) (see Table S2 in the supplemental material). A maximum likelihood tree was generated from core genome SNPs by using RAxML (66). Trees were visualized and annotated with Figtree (http://tree.bio.ed.ac.uk/software/figtree/) and Interactive Tree Of Life (67). Comparison of the MGE content and virulence factors of the isolates was assessed by BLAST analysis against Velvet *de novo* assemblies by using MGEs, virulence and antibiotic resistance genes previously reported to be present in the ST22 lineage (32, 68). Comparative genomics were carried out using Velvet *de novo* assemblies with contigs realigned against HO 5096 0412 by Mauve (69) and manually inspected with the Artemis comparison tool (70).

### Randomization tests.

To carry out randomization tests of phylogenetic clustering, we asked whether the mean evolutionary distance between members in a specified collection of isolates was significantly smaller than the mean distance between the same number of randomly chosen isolates. The reported *P* values were derived from a null distribution estimated from 10^6^ random samples of isolates, and all evolutionary distances were taken from the phylogeny depicted in Fig. 2A.

### Time scale of evolution.

We used the program BEAST v 1.7.5 (71) to estimate a dated phylogeny. To scale the rate of evolution, we constrained the tips of the phylogeny to the date that they were sampled in decimal years. If the day of the month was unknown, the first of the month was given as the date, and if the day and month were unknown, 1 June was used (see Fig. S1 in the supplemental material). Branch rates were drawn from an uncorrelated log normal distribution with mean rate assigned a uniform prior on the range 0 to 1. We assumed an HKY model of sequence evolution (72) with a gamma distribution of rate variation among sites. To model the relative node ages, we assumed either a constant or exponential population size coalescent tree prior. All other priors were assigned the defaults as specified in BEAUti v 1.7.5. We ran two separate Monte Carlo Markov chains (MCMCs) for each tree prior, using the maximum likelihood tree as a starting topology, until convergence in all parameters was reached and the burn-in was <10% of the entire run (~3 × 10^8^ iterations). The maximum clade credibility dated phylogeny (see Fig. S1) was generated from subsamples of the combined runs with burn-in removed. Estimates of the first date that ST22 *S. aureus* was found in dogs and cats were taken from the earliest node leading to a dog or cat isolate, respectively.

### Correlated evolution of virulence genes.

To test for correlated evolution between host the isolate was cultured from (human or companion animal) and virulence gene presence, we used the program BayesTraits (73), and the posterior sample of trees from our BEAST analysis using a constant coalescent tree prior. BayesTraits uses a continuous-time Markov model to estimate transition rates between the presence and absence of a virulence gene and between human and nonhuman hosts. We allowed the transition rates to evolve in either a correlated fashion (where the rate of change in one trait depends on the state found in the other trait) or independently. Posterior distributions of parameters were estimated from 4 × 10^7^ iterations of the MCMC with default priors. After discarding burn-in, the marginal likelihoods of the dependent and independent models were obtained using the harmonic mean estimator in Tracer v 1.5 (74) to yield Bayes factors for the competing models.

### Regression analysis.

We used discriminant analysis of principal components (DAPC) in order to identify putative single nucleotide polymorphisms (SNPs) with a role in host adaptation (75). DAPC identifies linear combinations of biallelic SNPs that best discriminate between different groups (in our case, human, dog, or cat host states). We retained only SNPs with a >1% frequency of the minor allele (a total of 897/6,979 SNPs) and transformed the data using the dudi.pca function in the R package *ade4*, keeping 10 principal components (PCs) for use in the DAPC analysis (76). In the DAPC analysis using the R package *adegenet*, we again retained 10 PCs representing 60% of the total genetic variation and kept one discriminant function. Visualization of variables that contribute to between-group discrimination identified five outliers (see Fig. S3 in the supplemental material). The SNPs are shown in Table S3 in the supplemental material. The ancestral state of the SNPs was reconstructed by mapping the locations of the SNPs back onto the phylogeny.

## SUPPLEMENTAL MATERIAL

Figure S1 Maximum clade credibility dated phylogeny of the core genomes of human and companion animal isolates of *S. aureus*. The scale axis represents the time in years and was estimated using the dated tip method in BEAST v 1.7.5. Human isolates are colored in black, dog isolates in red, and cat isolates in blue. Download Figure S1, TIFF file, 6.1 MB

Figure S2 The densities of genome sequences sampled from humans (red), dogs (yellow), and cats (blue), plotted against the first discriminant function. (i.e., the function of SNP data that best clusters the sequences by their host type.) The failure of this function to divide the data into three clear groups suggests a lack of SNPs that are found preferentially in a single host type. Results were obtained using discriminant analysis of principle components (75), retaining 10 PCs, but results were qualitatively unchanged when more or fewer PCs were retained. Download Figure S2, TIFF file, 0.4 MB

Figure S3 Variance contributions plot. Shown is the contribution of each SNP to the clustering of sequences by host type. The labeled SNPs make the largest contribution. Download Figure S3, TIFF file, 0.6 MB

Table S1 Isolates and associated metadata. Table S1, XLSX file, 0.1 MB.

Table S2 SNPs removed from alignment. Shown are the locations of regions in the HO 5096 0412 genome sequence encoding mobile genetic elements removed from the core SNPs used to generate the phylogeny Table S2, XLSX file, 0 1MB.

Table S3 SNPs showing correlation with host state (companion animals). Shown are the location and prevalence of, as well as further information about, the five SNPs showing a correlation with host state (companion animals). Table S3, XLSX file, 0 1MB.

## References

[1] Jevons MP 1961 “Celbenin”—resistant staphylococci. BMJ 1:124–125. 10.1136/bmj.1.5219.124

[2] Otto M 2012 MRSA virulence and spread. Cell. Microbiol. 14:1513–1521. 10.1111/j.1462-5822.2012.01832.x 22747834 PMC3443268

[3] Scott GM Thomson R Malone-Lee J Ridgway GL 1988 Cross-infection between animals and man: possible feline transmission of *Staphylococcus aureus* infection in humans? J. Hosp. Infect. 12:29–34. 10.1016/0195-6701(88)90119-3 2905371

[4] Cefai C Ashurst S Owens C 1994 Human carriage of methicillin-resistant *Staphylococcus aureus* linked with pet dog. Lancet 344:539–540. 10.1016/S0140-6736(94)91925-9 7914628

[5] Devriese LA Hommez J 1975 Epidemiology of methicillin-resistant *Staphylococcus aureus* in dairy herds. Res. Vet. Sci. 19:23–27 125447

[6] Lowder BV Guinane CM Ben Zakour NL Weinert LA Conway-Morris A Cartwright RA Simpson AJ Rambaut A Nübel U Fitzgerald JR 2009 Recent human-to-poultry host jump, adaptation, and pandemic spread of *Staphylococcus aureus*. Proc. Natl. Acad. Sci. U. S. A. 106:19545–19550. 10.1073/pnas.0909285106 19884497 PMC2780746

[7] Price LB Stegger M Hasman H Aziz M Larsen J Andersen PS Pearson T Waters AE Foster JT Schupp J Gillece J Driebe E Liu CM Springer B Zdovc I Battisti A Franco A Zmudzki J Schwarz S Butaye P Jouy E Pomba C Porrero MC Ruimy R Smith TC Robinson DA Weese JS Arriola CS Yu F Laurent F Keim P Skov R Aarestrup FM 2012 *Staphylococcus aureus* CC398: host adaptation and emergence of methicillin resistance in livestock. mBio 3(1):e00305-11. 10.1128/mBio.00305-11 22354957 PMC3280451

[8] Armand-Lefevre L Ruimy R Andremont A 2005 Clonal comparison of *Staphylococcus aureus* isolates from healthy pig farmers, human controls, and pigs. Emerg. Infect. Dis. 11:711–714. 10.3201/eid1105.040866 15890125 PMC3320358

[9] Paterson GK Larsen AR Robb A Edwards GE Pennycott TW Foster G Mot D Hermans K Baert K Peacock SJ Parkhill J Zadoks RN Holmes MA 2012 The newly described *mecA* homologue, *mecA*LGA251, is present in methicillin-resistant *Staphylococcus aureus* isolates from a diverse range of host species. J. Antimicrob. Chemother. 67:2809–2813. 10.1093/jac/dks329 22941897 PMC3494845

[10] García-Álvarez L Holden MT Lindsay H Webb CR Brown DF Curran MD Walpole E Brooks K Pickard DJ Teale C Parkhill J Bentley SD Edwards GF Girvan EK Kearns AM Pichon B Hill RL Larsen AR Skov RL Peacock SJ Maskell DJ Holmes MA 2011 Meticillin-resistant *Staphylococcus aureus* with a novel *mecA* homologue in human and bovine populations in the UK and Denmark: a descriptive study. Lancet Infect. Dis. 11:595–603. 10.1016/S1473-3099(11)70126-8 21641281 PMC3829197

[11] Walther B Wieler LH Vincze S Antão EM Brandenburg A Stamm I Kopp PA Kohn B Semmler T Lübke-Becker A 2012 MRSA variant in companion animals. Emerg. Infect. Dis. 18:2017–2020. 10.3201/eid1812.120238 23171478 PMC3557870

[12] Soares Magalhães RJ Loeffler A Lindsay J Rich M Roberts L Smith H Lloyd DH Pfeiffer DU 2010 Risk factors for methicillin-resistant *Staphylococcus aureus* (MRSA) infection in dogs and cats: a case-control study. Vet. Res. 41:55. 10.1051/vetres/2010028 20423695 PMC2879574

[13] Loeffler A Pfeiffer DU Lindsay JA Magalhães RJ Lloyd DH 2011 Prevalence of and risk factors for MRSA carriage in companion animals: a survey of dogs, cats and horses. Epidemiol. Infect. 139:1–10 20943000 10.1017/S095026881000227X

[14] Couto N Pomba C Moodley A Guardabassi L 2011 Prevalence of meticillin-resistant staphylococci among dogs and cats at a veterinary teaching hospital in Portugal. Vet. Rec. 169:72. 10.1136/vr.d1725 21502197

[15] Loeffler A Pfeiffer DU Lindsay JA Magalhães RJ Lloyd DH 2011 Prevalence of and risk factors for MRSA carriage in companion animals: a survey of dogs, cats and horses. Epidemiol. Infect. 139:1019–1028. 10.1017/S095026881000227X 20943000

[16] Loeffler A Boag AK Sung J Lindsay JA Guardabassi L Dalsgaard A Smith H Stevens KB Lloyd DH 2005 Prevalence of methicillin-resistant *Staphylococcus aureus* among staff and pets in a small animal referral hospital in the UK. J. Antimicrob. Chemother. 56:692–697. 10.1093/jac/dki312 16141276

[17] Weese JS Faires M Rousseau J Bersenas AM Mathews KA 2007 Cluster of methicillin-resistant *Staphylococcus aureus* colonization in a small animal intensive care unit. J. Am. Vet. Med. Assoc. 231:1361–1364. 10.2460/javma.231.9.1361 17975995

[18] Weese JS van Duijkeren E 2010 Methicillin-resistant *Staphylococcus aureus* and *Staphylococcus pseudintermedius* in veterinary medicine. Vet. Microbiol. 140:418–429. 10.1016/j.vetmic.2009.01.039 19246166

[19] Loeffler A Pfeiffer DU Lloyd DH Smith H Soares-Magalhaes R Lindsay JA 2010 Meticillin-resistant *Staphylococcus aureus* carriage in UK veterinary staff and owners of infected pets: new risk groups. J. Hosp. Infect. 74:282–288. 10.1016/j.jhin.2009.09.020 20080322

[20] Vincze S Stamm I Monecke S Kopp PA Semmler T Wieler LH Lübke-Becker A Walther B 2013 Molecular analysis of human and canine *Staphylococcus aureus* strains reveals distinct extended-host-spectrum genotypes independent of their methicillin resistance. Appl. Environ. Microbiol. 79:655–662. 10.1128/AEM.02704-12 23160118 PMC3553761

[21] Strommenger B Kehrenberg C Kettlitz C Cuny C Verspohl J Witte W Schwarz S 2006 Molecular characterization of methicillin-resistant *Staphylococcus aureus* strains from pet animals and their relationship to human isolates. J. Antimicrob. Chemother. 57:461–465. 10.1093/jac/dki471 16387748

[22] Coelho C Torres C Radhouani H Pinto L Lozano C Gómez-Sanz E Zaragaza M Igrejas G Poeta P 2011 Molecular detection and characterization of methicillin-resistant *Staphylococcus aureus* (MRSA) isolates from dogs in Portugal. Microb. Drug Resist. 17:333–337. 10.1089/mdr.2010.0080 21254810

[23] Ho PL Chow KH Lai EL Law PY Chan PY Ho AY Ng TK Yam WC 2012 Clonality and antimicrobial susceptibility of *Staphylococcus aureus* and methicillin-resistant *S. aureus* isolates from food animals and other animals. J. Clin. Microbiol. 50:3735–3737. 10.1128/JCM.02053-12 22895044 PMC3486263

[24] Zhang W Hao Z Wang Y Cao X Logue CM Wang B Yang J Shen J Wu C 2011 Molecular characterization of methicillin-resistant *Staphylococcus aureus* strains from pet animals and veterinary staff in China. Vet. J. 190:e125–e129. 10.1016/j.tvjl.2011.02.006 21382731

[25] Ferreira JP Anderson KL Correa MT Lyman R Ruffin F Reller LB Fowler VG Jr. 2011 Transmission of MRSA between companion animals and infected human patients presenting to outpatient medical care facilities. PLoS One 6:e26978. 10.1371/journal.pone.0026978 22102871 PMC3213111

[26] Rutland BE Weese JS Bolin C Au J Malani AN 2009 Human-to-dog transmission of methicillin-resistant *Staphylococcus aureus*. Emerg. Infect. Dis. 15:1328-1330. 10.3201/eid1508.081635 19751611 PMC2815967

[27] van Duijkeren E Wolfhagen MJ Box AT Heck ME Wannet WJ Fluit AC 2004 Human-to-dog transmission of methicillin-resistant *Staphylococcus aureus*. Emerg. Infect. Dis. 10:2235–2237. 10.3201/eid1012.040387 15663871 PMC3323405

[28] van Duijkeren E Wolfhagen MJ Heck ME Wannet WJ 2005 Transmission of a Panton-Valentine leucocidin-positive, methicillin-resistant *Staphylococcus aureus* strain between humans and a dog. J. Clin. Microbiol. 43:6209–6211. 10.1128/JCM.43.12.6209-6211.2005 16333133 PMC1317200

[29] Nienhoff U Kadlec K Chaberny IF Verspohl J Gerlach GF Schwarz S Simon D Nolte I 2009 Transmission of methicillin-resistant *Staphylococcus aureus* strains between humans and dogs: two case reports. J. Antimicrob. Chemother. 64:660–662. 10.1093/jac/dkp243 19608580

[30] Manian FA 2003 Asymptomatic nasal carriage of mupirocin-resistant, methicillin-resistant *Staphylococcus aureus* (MRSA) in a pet dog associated with MRSA infection in household contacts. Clin. Infect. Dis. 36:e26–e28. 10.1086/344772 12522764

[31] Ellington MJ Hope R Livermore DM Kearns AM Henderson K Cookson BD Pearson A Johnson AP 2010 Decline of EMRSA-16 amongst methicillin-resistant *Staphylococcus aureus* causing bacteraemias in the UK between 2001 and 2007. J. Antimicrob. Chemother. 65:446–448. 10.1093/jac/dkp448 20035019

[32] Holden MT Hsu LY Kurt K Weinert LA Mather AE Harris SR Strommenger B Layer F Witte W de Lencastre H Skov R Westh H Zemlicková H Coombs G Kearns AM Hill RL Edgeworth J Gould I Gant V Cooke J Edwards GF McAdam PR Templeton KE McCann A Zhou Z Castillo-Ramírez S Feil EJ Hudson LO Enright MC Balloux F Aanensen DM Spratt BG Fitzgerald JR Parkhill J Achtman M Bentley SD Nübel U 2013 A genomic portrait of the emergence, evolution, and global spread of a methicillin-resistant *Staphylococcus aureus* pandemic. Genome Res. 23:653–664. 10.1101/gr.147710.112 23299977 PMC3613582

[33] Harris SR Cartwright EJ Török ME Holden MT Brown NM Ogilvy-Stuart AL Ellington MJ Quail MA Bentley SD Parkhill J Peacock SJ 2013 Whole-genome sequencing for analysis of an outbreak of meticillin-resistant *Staphylococcus aureus*: a descriptive study. Lancet Infect. Dis. 13:130–136. 10.1016/S1473-3099(12)70268-2 23158674 PMC3556525

[34] Paterson GK Harrison EM Craven EF Petersen A Larsen AR Ellington MJ Török ME Peacock SJ Parkhill J Zadoks RN Holmes MA 2013 Incidence and characterisation of methicillin-resistant *Staphylococcus aureus* (MRSA) from nasal colonisation in participants attending a cattle veterinary conference in the UK. PLoS One 8:e68463. 10.1371/journal.pone.0068463 23869220 PMC3711812

[35] Köser CU Holden MT Ellington MJ Cartwright EJ Brown NM Ogilvy-Stuart AL Hsu LY Chewapreecha C Croucher NJ Harris SR Sanders M Enright MC Dougan G Bentley SD Parkhill J Fraser LJ Betley JR Schulz-Trieglaff OB Smith GP Peacock SJ 2012 Rapid whole-genome sequencing for investigation of a neonatal MRSA outbreak. N. Engl. J. Med. 366:2267–2275. 10.1056/NEJMoa1109910 22693998 PMC3715836

[36] Werckenthin C Schwarz S Westh H 1999 Structural alterations in the translational attenuator of constitutively expressed *ermC* genes. Antimicrob. Agents Chemother. 43:1681–1685 10390222 10.1128/aac.43.7.1681 PMC89343

[37] Harrison EM Paterson GK Holden G Larsen J Stegger M Larsen A Petersen A Skov R Christensen JM Zeuthen AB Heltberg O Harris S Zadoks R Parkhill J Peacock S Holmes MA 2013 Whole genome sequencing identifies zoonotic transmission of MRSA isolates with the novel *mecA* homologue *mecC*. EMBO Mol. Med. 5:509–515. 10.1002/emmm.201202413 23526809 PMC3628104

[38] Grundmann H Aanensen DM van den Wijngaard CC Spratt BG Harmsen D Friedrich AW European Staphylococcal Reference Laboratory Working Group 2010 Geographic distribution of *Staphylococcus aureus* causing invasive infections in Europe: a molecular-epidemiological analysis. PLoS Med. 7:e1000215. 10.1371/journal.pmed.1000215 20084094 PMC2796391

[39] Sakwinska O Giddey M Moreillon M Morisset D Waldvogel A Moreillon P 2011 *Staphylococcus aureus* host range and human-bovine host shift. Appl. Environ. Microbiol. 77:5908–5915. 10.1128/AEM.00238-11 21742927 PMC3165375

[40] Haenni M Saras E Châtre P Médaille C Bes M Madec JY Laurent F 2012 A USA300 variant and other human-related methicillin-resistant *Staphylococcus aureus* strains infecting cats and dogs in France. J. Antimicrob. Chemother. 67:326–329. 10.1093/jac/dkr499 22146878

[41] Lin Y Barker E Kislow J Kaldhone P Stemper ME Pantrangi M Moore FM Hall M Fritsche TR Novicki T Foley SL Shukla SK 2011 Evidence of multiple virulence subtypes in nosocomial and community-associated MRSA genotypes in companion animals from the upper midwestern and northeastern United States. Clin. Med. Res. 9:7–16. 10.3121/cmr.2010.944 20739580 PMC3064756

[42] Moodley A Espinosa-Gongora C Nielsen SS McCarthy AJ Lindsay JA Guardabassi L 2012 Comparative host specificity of human- and pig-associated *Staphylococcus aureus* clonal lineages. PLoS One 7:e49344. 10.1371/journal.pone.0049344 23166643 PMC3498157

[43] Nübel U Nachtnebel M Falkenhorst G Benzler J Hecht J Kube M Bröcker F Moelling K Bührer C Gastmeier P Piening B Behnke M Dehnert M Layer F Witte W Eckmanns T 2013 MRSA transmission on a neonatal intensive care unit: epidemiological and genome-based phylogenetic analyses. PLoS One 8:e54898. 10.1371/journal.pone.0054898 23382995 PMC3561456

[44] Huang SS Septimus E Kleinman K Moody J Hickok J Avery TR Lankiewicz J Gombosev A Terpstra L Hartford F Hayden MK Jernigan JA Weinstein RA Fraser VJ Haffenreffer K Cui E Kaganov RE Lolans K Perlin JB Platt R CDC Prevention Epicenters Program AHRQ DECIDE Network and Healthcare-Associated Infections Program 2013 Targeted versus universal decolonization to prevent ICU infection. N. Engl. J. Med. 368:2255–2265. 10.1056/NEJMoa1207290 23718152 PMC10853913

[45] Spoor LE McAdam PR Weinert LA Rambaut A Hasman H Aarestrup FM Kearns AM Larsen AR Skov RL Fitzgerald JR 2013 Livestock origin for a human pandemic clone of community-associated methicillin-resistant *Staphylococcus aureus*. mBio 4(4):e00356-13. 10.1128/mBio.00356-13 23943757 PMC3747577

[46] Gladysheva IP Turner RB Sazonova IY Liu L Reed GL 2003 Coevolutionary patterns in plasminogen activation. Proc. Natl. Acad. Sci. U. S. A. 100:9168–9172. 10.1073/pnas.1631716100 12878727 PMC170890

[47] de Haas CJ Veldkamp KE Peschel A Weerkamp F Van Wamel WJ Heezius EC Poppelier MJ Van Kessel KP van Strijp JA 2004 Chemotaxis inhibitory protein of *Staphylococcus aureus*, a bacterial antiinflammatory agent. J. Exp. Med. 199:687–695. 10.1084/jem.20031636 14993252 PMC2213298

[48] Rooijakkers SH Ruyken M Roos A Daha MR Presanis JS Sim RB van Wamel WJ van Kessel KP van Strijp JA 2005 Immune evasion by a staphylococcal complement inhibitor that acts on C3 convertases. Nat. Immunol. 6:920–927. 10.1038/ni1235 16086019

[49] Moodley A Stegger M Bagcigil AF Baptiste KE Loeffler A Lloyd DH Williams NJ Leonard N Abbott Y Skov R Guardabassi L 2006 *spa* typing of methicillin-resistant *Staphylococcus aureus* isolated from domestic animals and veterinary staff in the UK and Ireland. J. Antimicrob. Chemother. 58:1118–1123. 10.1093/jac/dkl394 17030517

[50] Walther B Monecke S Ruscher C Friedrich AW Ehricht R Slickers P Soba A Wleklinski CG Wieler LH Lübke-Becker A 2009 Comparative molecular analysis substantiates zoonotic potential of equine methicillin-resistant *Staphylococcus aureus*. J. Clin. Microbiol. 47:704–710. 10.1128/JCM.01626-08 19109463 PMC2650932

[51] Porrero MC Hasman H Vela AI Fernández-Garayzábal JF Domínguez L Aarestrup FM 2012 Clonal diversity of *Staphylococcus aureus* originating from the small ruminants goats and sheep. Vet. Microbiol. 156:157–161. 10.1016/j.vetmic.2011.10.015 22112857

[52] Walther B Wieler LH Friedrich AW Hanssen AM Kohn B Brunnberg L Lübke-Becker A 2008 Methicillin-resistant *Staphylococcus aureus* (MRSA) isolated from small and exotic animals at a university hospital during routine microbiological examinations. Vet. Microbiol. 127:171–178. 10.1016/j.vetmic.2007.07.018 17804179

[53] Lindsay JA 2014 Evolution of *Staphylococcus aureus* and MRSA during outbreaks. Infect. Genet. Evol. 21:548–553. 10.1016/j.meegid.2013.04.017 23665384

[54] Subbiah M Top EM Shah DH Call DR 2011 Selection pressure required for long-term persistence of blaCMY-2-positive IncA/C plasmids. Appl. Environ. Microbiol. 77:4486–4493. 10.1128/AEM.02788-10 21602382 PMC3127679

[55] Nagaev I Björkman J Andersson DI Hughes D 2001 Biological cost and compensatory evolution in fusidic acid-resistant *Staphylococcus aureus*. Mol. Microbiol. 40:433–439. 10.1046/j.1365-2958.2001.02389.x 11309125

[56] Nielsen KL Pedersen TM Udekwu KI Petersen A Skov RL Hansen LH Hughes D Frimodt-Møller N 2012 Fitness cost: a bacteriological explanation for the demise of the first international methicillin-resistant *Staphylococcus aureus* epidemic. J. Antimicrob. Chemother. 67:1325–1332. 10.1093/jac/dks051 22378682

[57] Björkman J Nagaev I Berg OG Hughes D Andersson DI 2000 Effects of environment on compensatory mutations to ameliorate costs of antibiotic resistance. Science 287:1479–1482. 10.1126/science.287.5457.1479 10688795

[58] Veterinary Medicines Directorate. 2009 Sales of antimicrobial products authorised for use as veterinary medicines, antiprotozoals, antifungals, growth promoters and coccidiostats, in the UK in 2007. Veterinary Medicines Directorate, New Haw, Surrey, United Kingdom

[59] Veterinary Medicines Directorate. 2007 Sales of antimicrobial products authorised for use as veterinary medicines, antiprotozoals, antifungals, growth promoters and coccidiostats, in the UK in 2006. Veterinary Medicines Directorate, New Haw, Surrey, United Kingdom

[60] Veterinary Medicines Directorate. 2006 Sales of antimicrobial products authorised for use as veterinary medicines, antiprotozoals, antifungals, growth promoters and coccidiostats, in the UK in 2005. Veterinary Medicines Directorate, New Haw, Surrey, United Kingdom

[61] Mateus A Brodbelt DC Barber N Stärk KD 2011 Antimicrobial usage in dogs and cats in first opinion veterinary practices in the UK. J. Small Anim. Pract. 52:515–521. 10.1111/j.1748-5827.2011.01098.x 21824147

[62] NHS Prescription Services. June 2013 Antibiotics national charts. NHS Prescription Services, NHS Business Services Authority, Newcastle upon Tyne, United Kingdom http://www.nhsbsa.nhs.uk/PrescriptionServices/Documents/PPDPrescribingAnalysisCharts/Antibiotics_Jun_2013_National.pdf PubMed

[63] Adriaenssens N Coenen S Versporten A Muller A Minalu G Faes C Vankerckhoven V Aerts M Hens N Molenberghs G Goossens H ESAC Project Group 2011 European surveillance of antimicrobial consumption (ESAC): outpatient macrolide, lincosamide and streptogramin (MLS) use in Europe (1997-2009). J. Antimicrob. Chemother. 66(Suppl 6):vi37–vi45. 10.1093/jac/dkq388 22096065

[64] Loeffler A Lloyd DH 2010 Companion animals: a reservoir for methicillin-resistant *Staphylococcus aureus* in the community? Epidemiol. Infect. 138:595–605. 10.1017/S0950268809991476 20056014

[65] Quail MA Kozarewa I Smith F Scally A Stephens PJ Durbin R Swerdlow H Turner DJ 2008 A large genome center’s improvements to the Illumina sequencing system. Nat. Methods 5:1005–1010. 10.1038/nmeth.1270 19034268 PMC2610436

[66] Stamatakis A Ludwig T Meier H 2005 RAxML-III: a fast program for maximum likelihood-based inference of large phylogenetic trees. Bioinformatics 21:456–463. 10.1093/bioinformatics/bti191 15608047

[67] Letunic I Bork P 2011 Interactive tree of life v2: online annotation and display of phylogenetic trees made easy. Nucleic Acids Res. 39:W475–W478. 10.1093/nar/gkr201 21470960 PMC3125724

[68] Zerbino DR Birney E 2008 Velvet: algorithms for de novo short read assembly using de Bruijn graphs. Genome Res. 18:821–829. 10.1101/gr.074492.107 18349386 PMC2336801

[69] Darling AC Mau B Blattner FR Perna NT 2004 Mauve: multiple alignment of conserved genomic sequence with rearrangements. Genome Res. 14:1394–1403. 10.1101/gr.2289704 15231754 PMC442156

[70] Carver TJ Rutherford KM Berriman M Rajandream MA Barrell BG Parkhill J 2005 ACT: the Artemis comparison tool. Bioinformatics 21:3422–3423. 10.1093/bioinformatics/bti553 15976072

[71] Drummond AJ Suchard MA Xie D Rambaut A 2012 Bayesian phylogenetics with BEAUti and the BEAST. Mol. Biol. Evol. 29:1969–1973. 10.1093/molbev/mss075 22367748 PMC3408070

[72] Hasegawa M Kishino H Yano T 1985 Dating of the human-ape splitting by a molecular clock of mitochondrial DNA. J. Mol. Evol. 22:160–174. 10.1007/BF02101694 3934395

[73] Pagel M 1994 Detecting correlated evolution on phylogenies: a general method for the comparative analysis of discrete characters. Proc. R. Soc. Lond. B Biol. Sci. 255:37–45. 10.1098/rspb.1994.0006

[74] Drummond AJ Rambaut A 2007 BEAST: bayesian evolutionary analysis by sampling trees. BMC Evol. Biol. 7:214. 10.1186/1471-2148-7-214 17996036 PMC2247476

[75] Jombart T Devillard S Balloux F 2010 Discriminant analysis of principal components: a new method for the analysis of genetically structured populations. BMC Genet. 11:94. 10.1186/1471-2164-11-94 20950446 PMC2973851

[76] Dray S Dufour A 2007 The ade4 Package: implementing the duality diagram for ecologists. J. Stat. Softw. 22 www.jstasoft.org/v22/i04

