# Supplementary figures and images for: A Shared Population of Epidemic Methicillin-Resistant Staphylococcus aureus 15 Circulates in Humans and Companion Animals
Source: mBio. 2014 May 13;5(3):e00985-13. doi: 10.1128/mBio.00985-13 (PMC4030480; doi:10.1128/mBio.00985-13)

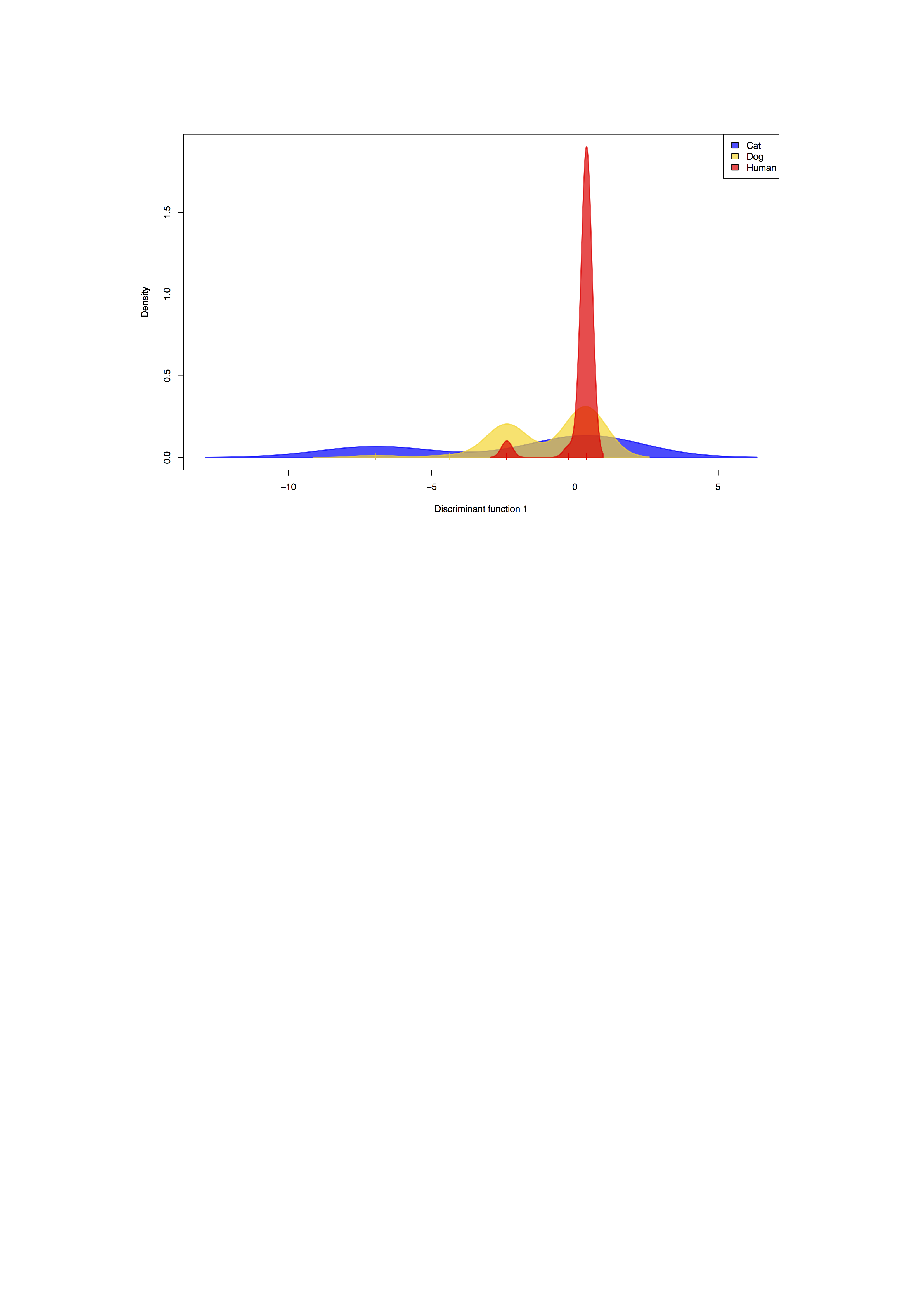

Supplement: Figure S2 — The densities of genome sequences sampled from humans (red), dogs (yellow), and cats (blue), plotted against the first discriminant function. (i.e., the function of SNP data that best clusters the sequences by their host type.) The failure of this function to divide the data into three clear groups suggests a lack of SNPs that are found preferentially in a single host type. Results were obtained using discriminant analysis of principle components (75), retaining 10 PCs, but results were qualitatively unchanged when more or fewer PCs were retained. Download [file mbo002141819sf02.tif]

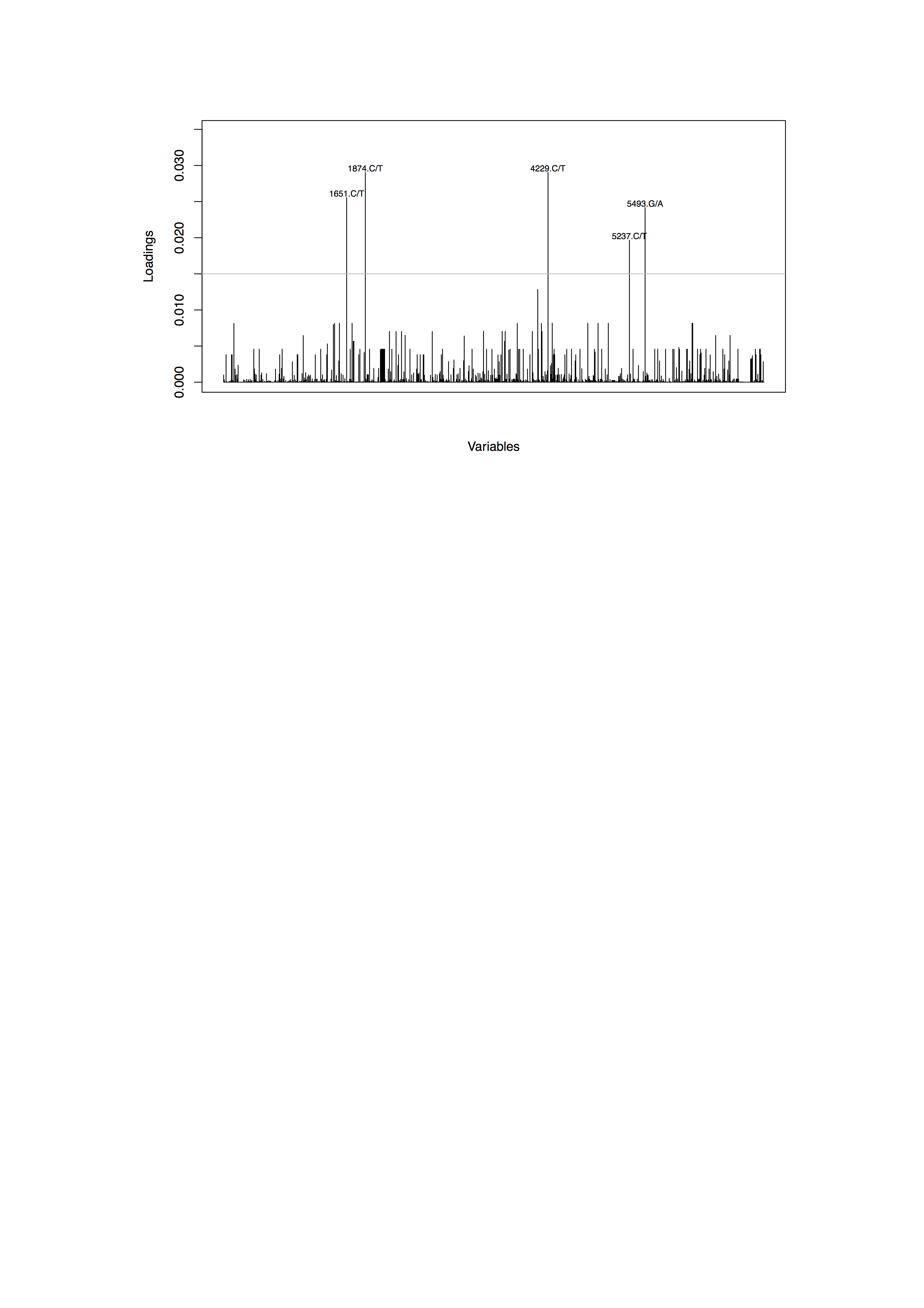

Supplement: Figure S3 — Variance contributions plot. Shown is the contribution of each SNP to the clustering of sequences by host type. The labeled SNPs make the largest contribution. Download [file mbo002141819sf03.tif]
